# Supplementary material for: Silicon-Based Superslippery/Superhydrophilic Striped Surface for Highly Efficient Fog Harvesting
Source: Materials (Basel). 2023 Aug 2;16(15):5423. doi: 10.3390/ma16155423 (PMC10419386; doi:10.3390/ma16155423)
Supplement: Supplementary file 1 [file materials-16-05423-s001.zip › materials-2514108-supplementary.pdf]

Supplementary Information

Silicon-Based Superslippery /Hydrophilic Patterned Surfaces for Highly Efficient Fog Harvesting

Xiang Ji, Shunxu Shuai, Shuai Liu, Yuyan Weng, Lu You, Liang Fang, Fengang Zheng*,

School of Physical Science and Technology, Jiangsu Key Laboratory of Thin Films, Soochow University, Suzhou 215006, China

*Corresponding author: zhfg@suda.edu.cn

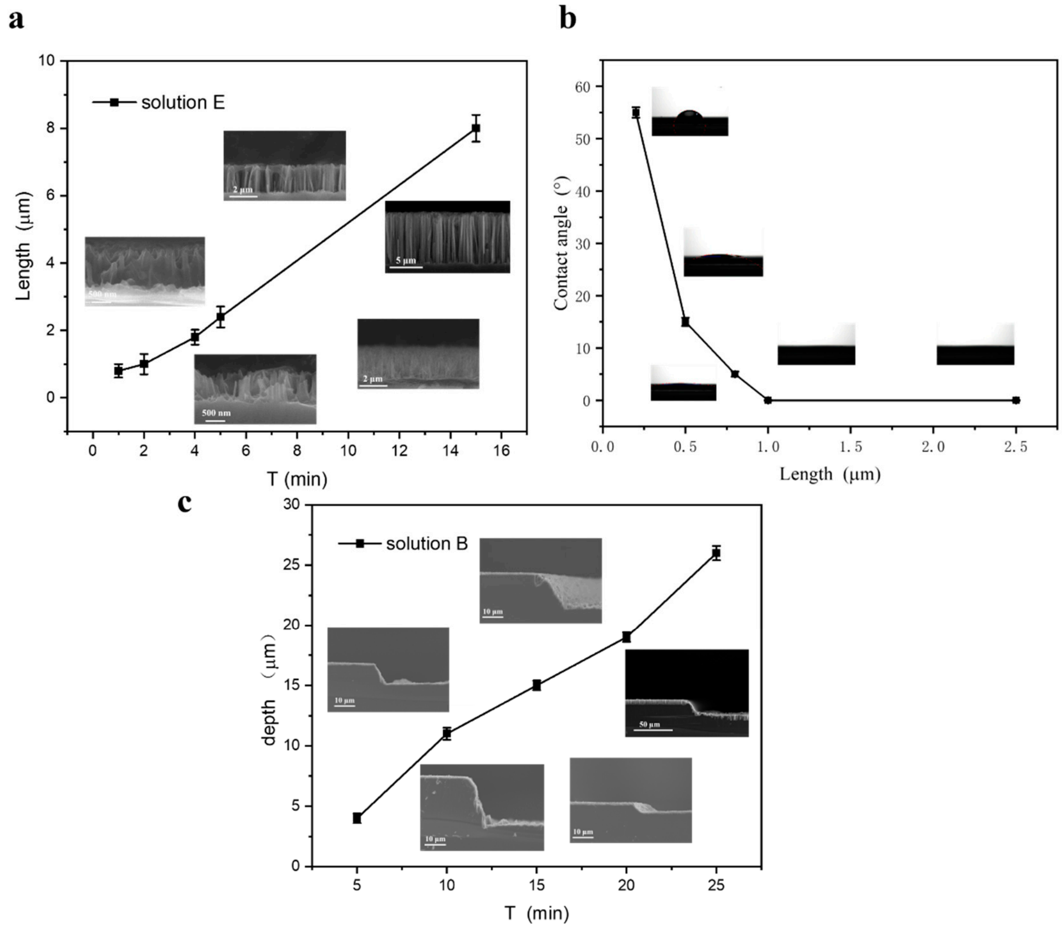

Fig. S1. (a) Effect of etching time on nanowire length (b) Effect of nanowire length on wettability (c) Effect of etching time on trench depth

As shown in Fig. S1a, the length of nanowires gradually increases with the increase of etching time in solution E, and the length is 1 μm at 1 min of etching time and 8 μm at 15 min. The length of nanowires affects the wettability of the surface, as shown in Fig. S1b, when the length of nanowires is 200 nm, the contact angle is about 55°, and it still shows hydrophilicity. As the nanowires grow, the contact angle becomes smaller and smaller. After 1 μm, the surface shows superhydrophilicity and remains constant from then on. As shown in Figure S1c, we were also able to easily change the depth of the grooves by regulating the etching time in solution B. The longer the etching time, the deeper the groove.

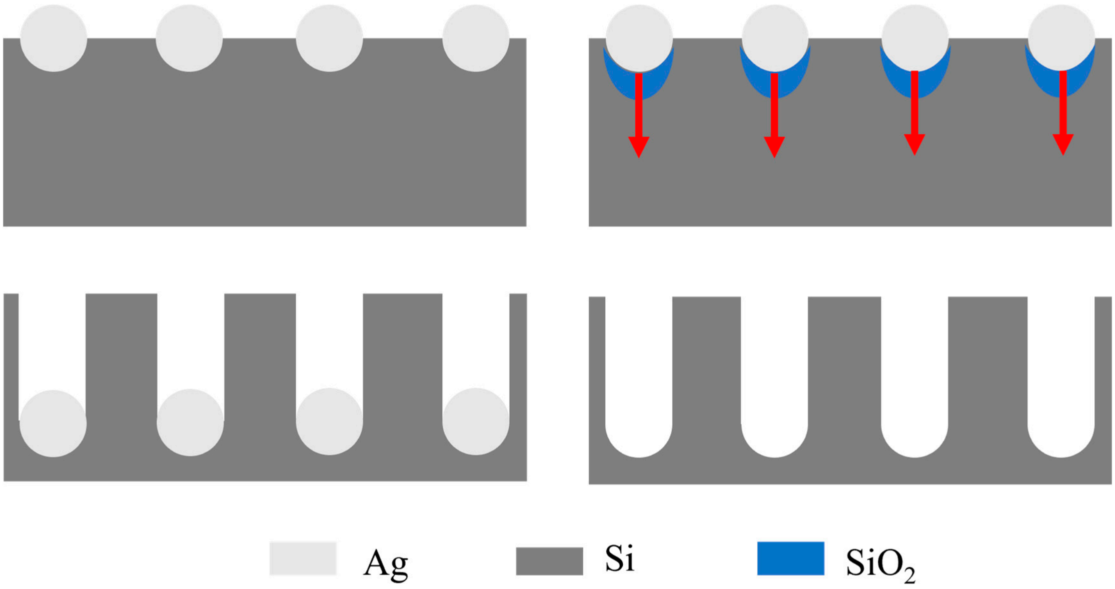

Fig. S2. Schematic diagram of silver-assisted chemical etching

Silicon wafers are etched using silver-assisted chemical etching, and silver particles are first deposited on the surface with silver nitrate solution. After the silver particles are in contact with silicon, since the work function of silver is greater than that of silicon, the two form a Schottky contact, a hole accumulation area is formed at the interface, and a SiO_2 layer is formed under the action of H_2O_2. Due to the presence of HF in the solution, the oxide layer is quickly dissolved, and the nano-silver particles sink. As the oxidation-reduction reaction proceeds, the longitudinal movement of the nano-silver particles deepens. Finally, after cleaning with a certain concentration of HNO_3 solution, Si nanowires are formed on the pore wall.

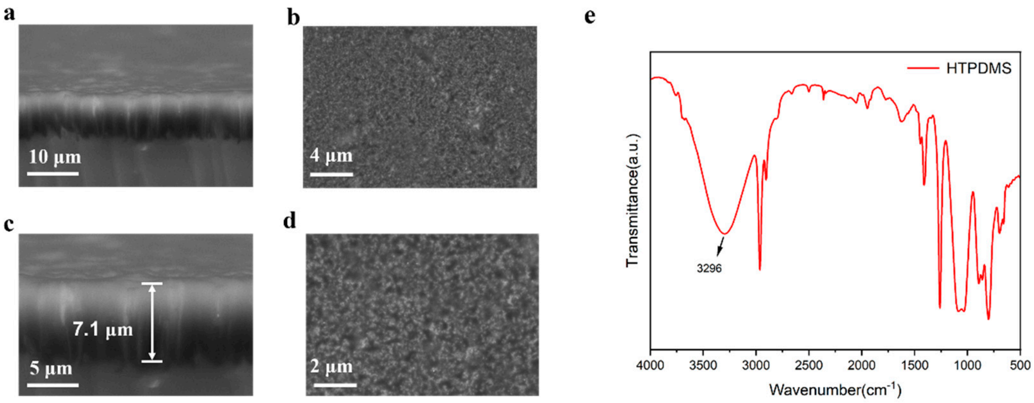

Fig. S3. (a,c) SEM cross section image of superhydrophilic region after filling lubricant; (b,d) SEM surface image of superhydrophilic region; (e) FTIR spectra of the HTPDMS

As can be seen from the figure, HTPDMS was successfully injected into the nanowire structure, and the surface was covered by an oil film. FTIR spectrum of the HTPDMS is shown in Fig. S3. The existence of OH absorption band at 3296 cm^{-1} in the sample.

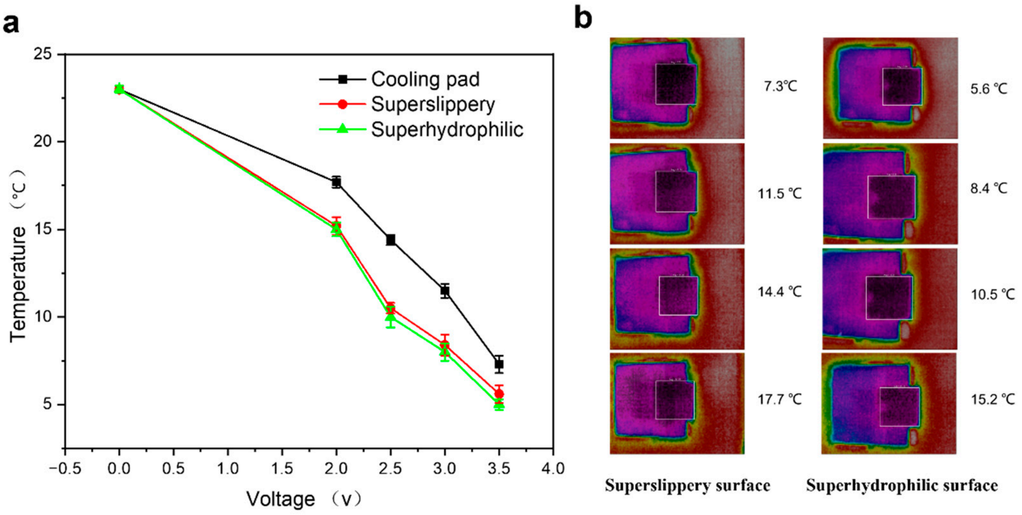

Fig. S4. (a) The relationship between cooling voltage and cooling pad temperature (b)Infrared thermography of superhydrophilic and superslippery surfaces under different condensation conditions

As shown in Fig. S4, with the increase of refrigeration voltage, the temperature of the cooling pad gradually decreases. Under the same refrigeration condition, the temperature of the superhydrophilic surface is slightly lower than that of the superslippery surface, which is due to the existence of lubricants on the super slippery surface, which reduces its heat transfer coefficient.

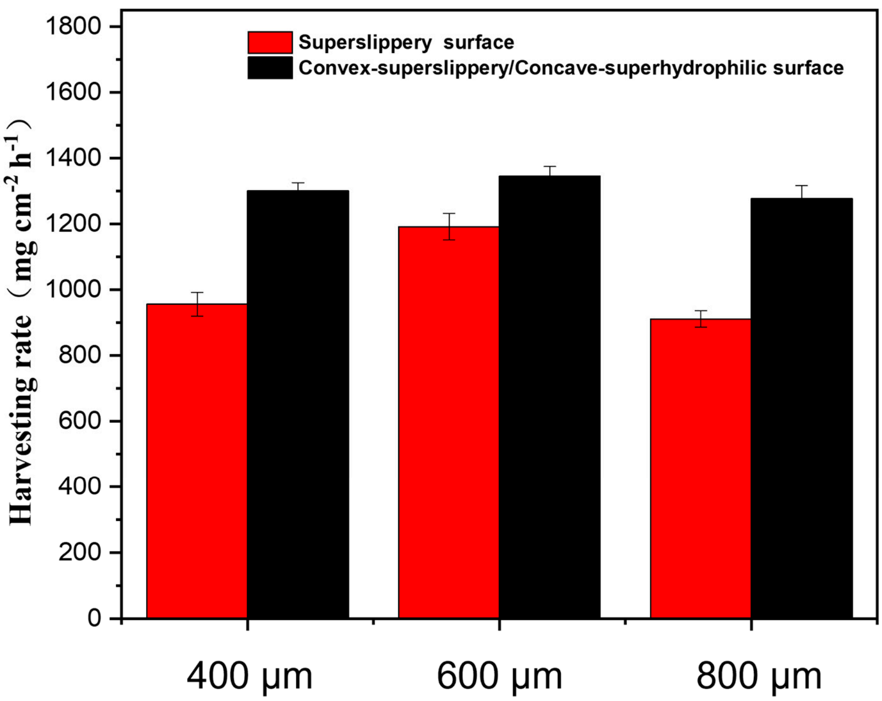

Fig. S5. Fog-harvesting rate of superslippery surface and convex-superslippery/concave-superhydrophilic surface with different stripe widths under the condition of room temperature

As shown in Fig. S5, the fog-harvesting rates of two different tables with different stripe widths showed a consistent pattern. The worst performance was observed for a stripe width of 800um, followed by 400um, while the highest fog-harvesting rate was 1192 mg · cm^2 · h^{-1} and 1345 mg · cm^{-2} · h^{-1} for a stripe width of 600um, respectively.

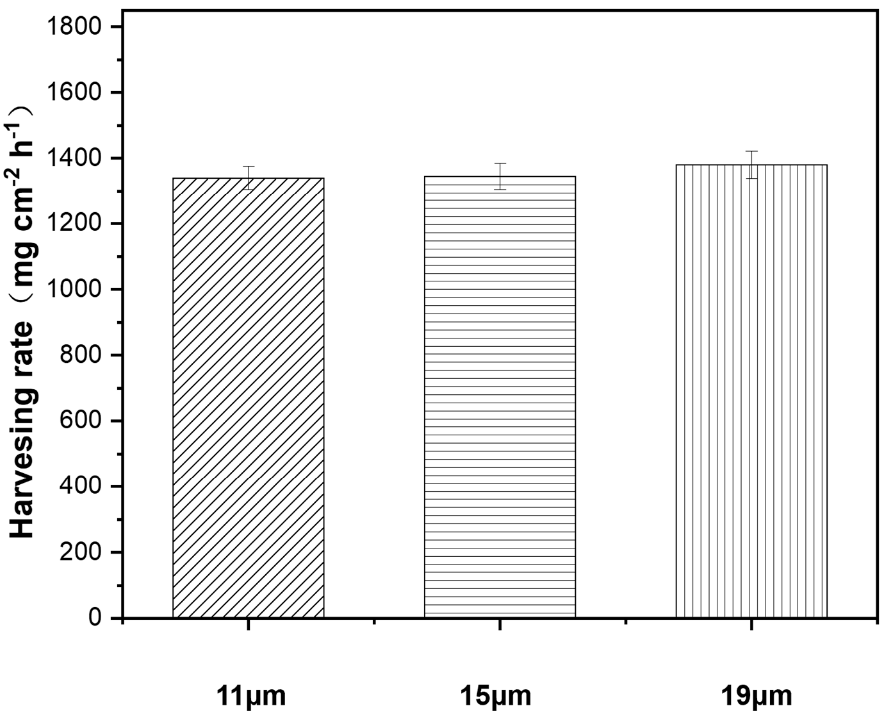

Fig. S6. Fog-harvesting rate of convex-superslippery/concave-superhydrophilic surface with different groove depths of 600 μm stripe width at room temperature

As shown in Fig. S6, the fog-harvesting rate was around 1350 mg · cm^{-2} · h^{-1} when the groove depths were 11, 15, and 19 μm, respectively, i.e., the effect of groove depth on fog-harvesting rate was negligible.

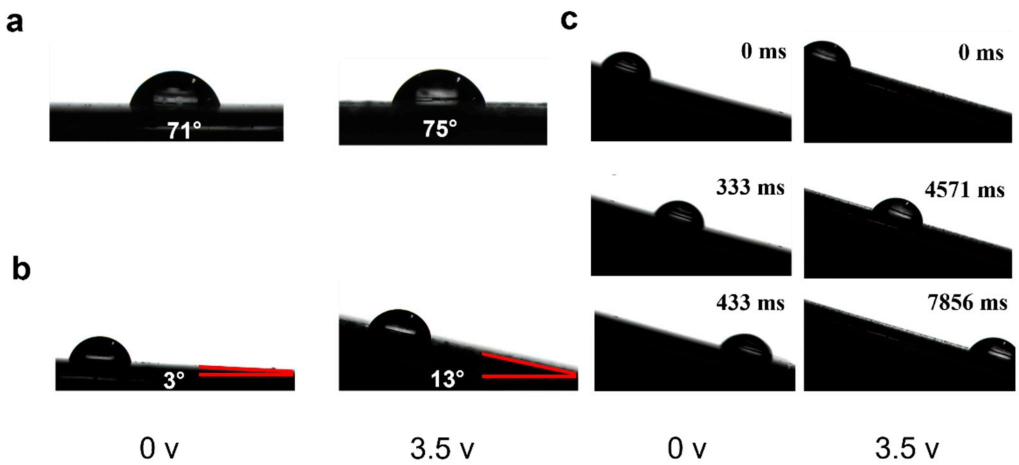

Fig. S7 (a) Contact angles and (b) Sliding angle of superslippery surfaces under different

refrigeration voltages (c) Under different refrigeration voltages, at an inclination angle of 15°, the state of a 5 μl droplet sliding on a super-slippery surface parallel to the direction of the stripes

As shown in Fig. S7, compared with the room temperature environment, under the cooling condition of 3.5v, both the contact angle and the sliding angle of the droplet increased. The contact angle changed from 71 ° to 75 °, while the sliding angle changed from 3 ° to 13 °. The time required for the droplet to slide from the top to the bottom of the slope also changed from 433ms to 7856ms. These phenomena all indicate that under cooling conditions, the sliding resistance of the superslippery surface will increase, which will affect the fog-harvesting rate.

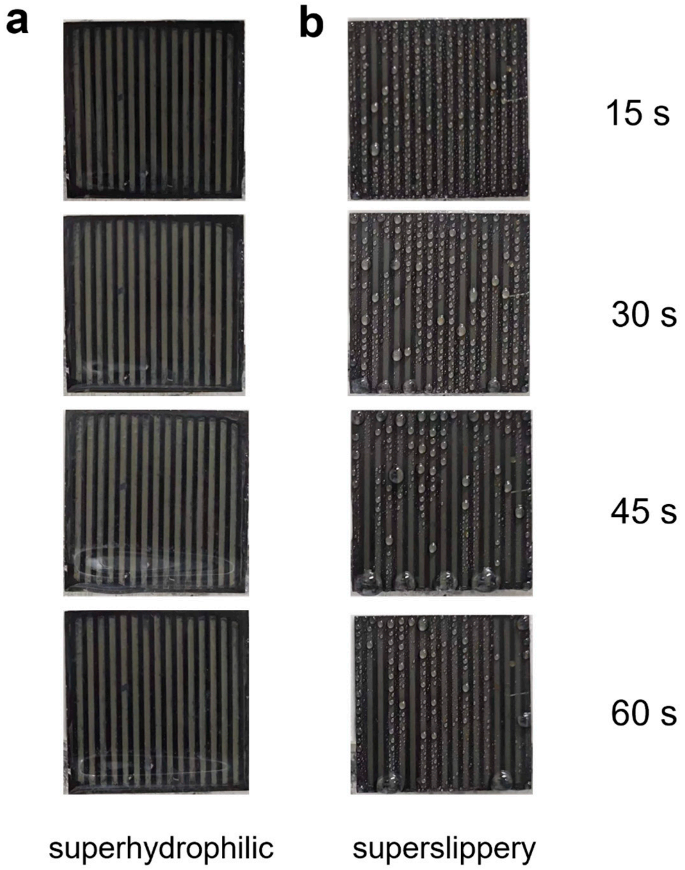

Fig. S8. Water droplet motion state on two different surfaces during fog harvesting under 3.5 v cooling conditions

We also explored the effect of cooling on superhydrophilic and superslippery surfaces during fog harvesting. As shown in Fig. S8, under the condition of a refrigeration voltage of 3.5 v, the collection rate of fog on the superhydrophilic surface was significantly higher than that at room temperature, and water droplets began to drip after 45 s. Under the same conditions, the movement behavior of droplets on the superslippery surface is not significantly improved compared with that at room temperature, and the droplets still drop after 45s.

Video Captions

Video S1

A 5 μL water droplet slides along parallel stripes on a HTPDMS infused superslippery surfaces with width and spacing of 0.6 mm at an inclination angle of 15°.

Video S2

A 5 μL water droplet slides along vetrical stripes on a HTPDMS infused superslippery surfaces with width and spacing of 0.6 mm at an inclination angle of 15°.

Video S3

A 5 μL water droplet slides along parallel stripes on a HTPDMS infused superslippery/superhydrophilic striped surface with width and spacing of 0.6 mm at an inclination angle of 15°.

Video S4

A 5 μL water droplet slides along vertical stripes on a HTPDMS infused superslippery/superhydrophilic striped surface with width and spacing of 0.6 mm at an inclination angle of 15°.

Video S5

Fog harvest on superslippery surfaces with array of stripes with width of 0.6 mm.

Video S6

Fog harvest on superslippery/hydrophilic striped surface with a strip width of 0.6 mm.
